# A qualitative evaluation of occupational therapy-led work rehabilitation for people with inflammatory arthritis: Patients’ views

**DOI:** 10.1177/0308022616672666

**Published:** 2016-11-21

**Authors:** Yeliz Prior, Aparna Evangelina Amanna, Sarah Jane Bodell, Alison Hammond

**Affiliations:** 1 Research Fellow, Centre for Health Sciences Research, University of Salford, UK; 2 Advanced Clinical Specialist Occupational Therapist, Mid Cheshire NHS Trust Hospitals, Leighton Hospital, Crewe, UK; 3 Research Assistant, Centre for Health Sciences Research, University of Salford, UK; 4 Senior Lecturer in Occupational Therapy, School of Health Sciences, University of Salford, UK; 5 Professor of Rheumatology, Rehabilitation, Centre for Health Sciences Research, University of Salford, UK

**Keywords:** Work rehabilitation, qualitative, rheumatology

## Abstract

**Introduction:**

This qualitative study, nested in a pilot feasibility randomised controlled trial, explored the views of working people with inflammatory arthritis on the impact of a work rehabilitation programme received.

**Method:**

Thirty-two participants, drawn from the 55 participants in the associated randomised controlled trial, were recruited from secondary care in the United Kingdom. Semi-structured telephone and face-to-face interviews were conducted at six (*n* = 32) and nine months follow-up (*n* = 31). Interviews were audio-recorded, transcribed, and analysed using a constant comparative approach, under the theoretical framework of critical realism.

**Findings:**

Three overarching themes emerged: (1) intervention group participants valued the work rehabilitation programme received, and highlighted the benefits of occupational therapy; (2) control group participants reported no benefits in relation to the written work advice pack, and lacked future aspirations to stay employed; (3) the majority of participants reported not reading the written work advice pack provided, which was the only work advice received by the control group.

**Conclusion:**

Working people with inflammatory arthritis highly valued the practical support received from the therapists, and emphasised the value of the therapeutic relationship in the rehabilitation process. A tailor-made work rehabilitation programme, which incorporates cognitive-behavioural strategies into patient education, may help to reduce work instability in people with inflammatory arthritis, and increase their perceived self-efficacy.

## Introduction

Musculoskeletal conditions (MSCs) represent 40% of all days lost due to work-related ill health in Great Britain (Health and Safety Executive, 2015). Amongst these MSCs, work loss is commonly reported in people with rheumatoid arthritis (RA), a systemic inflammatory condition that affects 1% of the world population. Prior to leaving paid work, productivity loss and sickness leave is common in employed people with RA, which is associated with symptoms of pain, fatigue, and impaired morning function (Strand and Khanna, 2010). Frequent sick leave and productivity loss is indicative of work instability, which occurs when there is a mismatch between the individual’s functional capacity and work demands (Gilworth et al., 2003). Work instability leads to premature work cessation (Gilworth et al., 2003), and thus is an important marker for health professionals working with individuals with RA to help to prevent work disability. Prevention of work disability is particularly important as work is often not a treatment goal for those who cease to be employed; therefore, once people with RA stop work, they are unlikely to start again (Hammond, 2004). Work interventions for job retention might therefore be more effective than strategies to regain it (Prior and Hammond, 2014a; Prior et al., 2015).

Work rehabilitation interventions aimed at job retention require the ability to identify people with early RA; however, a diagnosis of RA is a lengthy process as many rheumatic conditions are chronic systemic inflammatory diseases and share overlapping symptoms, as well as laboratory markers (Imboden et al., 2013). Whilst going through diagnostic assessment, patients are initially diagnosed with inflammatory arthritis (IA) prior to a definitive RA diagnosis. IA is an umbrella term for a group of conditions that affect the immune system in such a way that the body’s defence system begins attacking its own tissues, causing pain, stiffness, and joint damage. Therefore, IA is characterised by stiffness, pain, swelling, and tenderness of the joints and surrounding ligaments and tendons (Dewing, 2015; Gottlieb et al., 2008). RA and psoriatic arthritis (PsA) are amongst the most common IA conditions.

Work rehabilitation is defined as ‘whatever helps someone with a health problem to stay at, return to and remain in work’ (Diracoglu et al., 2008). The National Institute for Health and Care Excellence (NICE) guidelines on managing long-term sickness and incapacity for work emphasise referral to occupational therapy for patients with RA who are experiencing limitations in daily activities and work (NICE, 2016).

This qualitative study^1^ investigating the vocational, clinical, and cost-effectiveness of occupational therapy-led work rehabilitation in working people with IA aimed to explore participants’ views of the work rehabilitation programme received and to identify the impact of it on their life and work status.

## Literature review

Work status, health, and income are strongly related and work disability is associated with adverse health and social outcomes (Bansback et al., 2012; Strand and Khanna, 2010; Uhlig, 2010). In recent years, the introduction of biological agents targeting inflammatory cytokines such as tumour necrosis factor alpha (TNF-alpha) have been increasingly prescribed for a variety of inflammatory conditions, particularly for IA. Anti-tumour necrosis factor (Anti-TNF) therapy is thought to have revolutionised the treatment of IA (Arthritis Research UK, 2016); however, even people treated with Anti-TNF therapy continue to face challenges in their working lives, despite reporting improvements in function. Thus, health professionals’ input to support work participation, particularly to help with social and psychological issues, remains important (Van der Meer et al., 2011).

Studies conducted in the United States (USA) have shown that timely, patient-centred work rehabilitation assists with work retention and reducing work disability in people with rheumatic conditions (Allaire and Keysor, 2009; Allaire et al., 2011). In the United Kingdom (UK), Macedo et al. (2009) demonstrated that comprehensive occupational therapy and work interventions are effective for improving functional and work-related outcomes in employed people with RA. An ergonomic work place intervention was found to decrease work difficulties and improve physical functioning and pain in employed people with RA (Baldwin et al., 2012). However, despite growing evidence of work rehabilitation interventions for employed people with rheumatic conditions, there is a paucity of research to evaluate the impact of occupational therapy-led work rehabilitation in people with inflammatory arthritis (Prior and Hammond, 2014).

## Method

### Study design

Qualitative methodology was chosen to elicit working people with IA’s views of the work rehabilitation programme received. Qualitative research offers an in-depth exploration of individuals’ experiences, accounts for the complexity of the context, and takes different perspectives on board (Sofaer, 1999). In addition, the use of mixed methodology in feasibility randomised controlled trials (RCTs) provides a support for best evidence as they aim to understand how the intervention(s) work and evaluate the design in preparation of the full trial (O’Cathain et al., 2015).

### Ethics

The study was approved by the NRES Committee East Midlands, Nottingham. All participants were provided with the study information sheet and written consent was obtained. Confidentiality and anonymity were addressed by using pseudonyms and aggregating demographic data.

### Participants

Initially, participants were recruited into the RCT by research nurses from rheumatology out-patient departments in five National Health Service (NHS) hospitals and one Primary Care-based musculoskeletal service. People with IA (specifically early IA, RA, or PsA) were recruited. Inclusion criteria were: aged 18 years and over; able to read, write, and understand English; in paid work (full or part time); with job concerns because of arthritis. People were excluded who were: on extended sick leave (3 months or more); unemployed (including not normally in paid employment or a student); planning to retire or take early retirement (through choice or ill health) within the next 12 months; already receiving or awaiting work rehabilitation services; planning to move out of area; or expecting joint replacement surgery in the next 6 months. For this qualitative study, the researcher was provided with the contact information of the participants who consented to take part in the interviews at the time of enrolment to the RCT, following the treatment delivery, and on completion of the 6-month follow-up questionnaires. Participants were mailed a patient information sheet and reminded about the qualitative interviews at this point. The researcher then contacted the participants by telephone to arrange an appropriate time to hold the interview, and verbal consent was obtained.

### The work rehabilitation programme

#### The intervention

Participants in the intervention group received a work rehabilitation intervention, modified from a programme developed by Allaire et al. (2003) in the US, which was adapted for use in the UK by Hammond et al. (2011). The intervention consisted of three 1.5-hour one-to-one meetings with a rheumatology occupational therapist, plus a 30-minute telephone review to evaluate actions taken. An optional 1.5 hours of further contact was provided for those with more serious work problems such as those identified as being at high risk of work disability. The Work Experience Survey – Rheumatic Conditions (WES-RC) (Allaire and Keysor, 2009) was used to identify participants’ work problems (physical, psychological, environmental (physical/social/managerial) through a semi-structured interview conducted by a rheumatology occupational therapist.

#### Written work advice pack

All participants were mailed a self-help work information pack, which included information on: managing work problems; how to access existing work rehabilitation support; and employment rights for working people with disabilities. The control group received the written work information pack only, as per the usual treatment available from the NHS (Prior and Hammond, 2014).

## Data collection

Semi-structured telephone and face-to-face interviews were conducted with study participants by an independent (not involved in the delivery of the RCT), trained qualitative researcher at 6 and 9 months following the work programme delivery. Interview questions were devised in an order where open, general questions were asked prior to any prompts to avoid bias. The interviewer was blinded to the treatment group allocation to reduce potential interviewer bias, with an understanding that unblinding may occur at some point during the interviews.

The interviewer remained neutral throughout the interviews, moderating their tone of voice and refraining from expressing opinions. During face-to-face interviews, the interviewer also endeavoured to moderate their facial expressions and body language to ensure participants’ views were not influenced. Participants were, however, challenged when thought to be giving socially acceptable answers that may be false, and encouraged to reveal their true views and feelings. This was achieved by building a rapport with participants to cultivate their trust in the researcher, which is an important element in qualitative data collection.

### Telephone interviews at six months

Following the completion of the work rehabilitation programme, all participants were interviewed at 6 months by telephone to ascertain their views of the work rehabilitation intervention or the written advice received in this trial. Conducting these interviews at 6 months was deemed appropriate to allow sufficient time for participants to adopt any behavioural strategies and/or to implement any ergonomic changes at their workplace as a result of the work rehabilitation intervention and/or the written work advice received in this study. Telephone interviews were chosen for convenience, as employed participants could only be interviewed in the evenings or weekends to avoid taking time off work. The semi-structured telephone interview schedule was devised by the trial management group, which included patient research partners and work rehabilitation experts. The schedule consisted of questions such as: ‘In your view, what factors have enabled you to stay in work?’ or, if the person was no longer in work, ‘In your view, what were the reasons for you stopping work?’ Prompts were used, as necessary, to explore further, such as: ‘Have you made any practical changes?’ and ‘Were you able to discuss your condition with your employer?’ (see online appendices at http://bjo.sagepub.com/content/by/supplemental-data.)

### Face-to-face interviews

In order to obtain rich and in-depth data, at 6 months post-intervention, semi-structured face-to-face interviews (lasting 30–60 minutes) were conducted with a sub-sample of participants who agreed to meet to further explore the themes that emerged from the telephone interviews. The interviewer was unblinded at this stage of the interviews, and purposeful sample participants with equal numbers from each of the treatment groups were drawn with a range of age, gender, socio-demographic, health, and socio-economic statuses to account for a variety in the sample. Interviews took place in participants’ own homes or private rooms at the host hospital, as per the participant’s preference.

### Closing telephone interviews at 9 months

Participants were also followed up by conducting closing interviews at 9 months by telephone, to ascertain if there were any changes to their work status and explore the long-term impact of the work intervention and/or the written work advice received in this study. The closing interviews lasted between 15 and 20 minutes and marked the end of participants’ involvement in the study.

### Data management and analysis

All interviews were audio-recorded and transcribed verbatim, with names replaced by codes to maintain anonymity. Any field notes taken to remember and record the behaviours, activities, events, and other features of any observations made during the interview was also transcribed and used to help with the interpretation of the transcripts.

The Qualitative Research Data Analysis Software QSR NVivo 10 facilitated the analysis process. Data collected at each time point were stratified by participant in NVivo, identifying the time points for each transcription with annotations. Thematic analysis using a constant comparative analysis method (CCA) was employed using a critical realist theoretical framework. This theory supports the concept of ontological realism; in other words, our understanding of ‘reality’ (including disease, biological factors, socio-demographic context) is subject to our own perspectives and standpoint (Mythen and Walklate, 2006; Williams and Department of Sociology UoW, 2014). As disability occurs at a participation level and is associated with personal and environmental factors (Prior, 2013), a critical realist perspective was chosen to provide a wider angle to the interpretation process by encouraging the researcher to take biopsychosocial factors into account. The CCA (Charmaz, 2006) method was chosen as an appropriate approach to provide a theoretical sampling (what group or subgroups the researcher turns to next to collect data) to inform the design of a future, definitive RCT. Following this approach, codes were identified by reading the transcripts several times, making initial notes of special incidents, and constant comparison of codes to identify and explore relationships, refine concepts, and identify priorities, resulting in coherent themes that encapsulate the patterns in the data collected.

In qualitative research, the trustworthiness of interpretations and findings are dependent on the justification of the methods by which these were obtained (Mauthner and Doucet, 2003). Confirmability of the emerging themes was supported by two researchers analysing the data independently, and then comparing, discussing, and agreeing main themes, followed by a third researcher reviewing a random selection of the interview transcripts and their analyses, thus enabling a cross-examination of the confirmability of the interpretations. Once researchers agreed on the main themes, the final report was sent to participants to ensure the interpretation of the data was neutral, credible, and reflected their views, in order to support the trustworthiness of the findings.

## Findings

### Participants

Of the 55 participants in the RCT, 32 people were interviewed at 6 months by telephone, following the work rehabilitation intervention delivery and the receipt of the written work information pack. As data saturation was reached, and an equal numbers of participants (*n* = 16 from each treatment group) were interviewed, remaining trial participants who consented to take part in the qualitative study were not interviewed. Table 1 shows the basic demographic and health characteristics of the trial participants in terms of people interviewed in this qualitative study compared to those who were not interviewed (32 compared with 23). There were no statistically significant differences between the two groups in terms of their socio-demographic and health characteristics.

**Table 1.** Comparison of the baseline demographic and health characteristics of the randomised controlled trial (RCT) participants who were interviewed in this study with those who were not interviewed (*n* = 55).

	Participants not interviewed *n* = 23	Participants interviewed *n* = 32	*P* value
Age (years) at enrolment to RCT^a^			
Mean (SD)	49.61 (9.4)	49.38 (8.5)	0.92
Gender *n* (%)^b^			
Male	4 (17.4)	9 (28.1)	
Female	19 (82.6)	23 (71.9)	0.36
Multidimensional HAQ score^a,c,d^			
0–3: Mean (SD)			
Functional status score	0.6 (0.4)	0.7 (0.4)	0.76
Psychological score	0.9 (0.6)	1.0 (0.6)	0.43
Symptom severity and Health status^a,e^ (0–100)			
Pain due to condition	46.9 (28.9)	49.13 (20.2)	0.74
Pain in the hands and wrists	44.8 (27.5)	56.5 (25.2)	0.11
Unusual fatigue	59.3 (28.5)	59.4 (25.6)	0.99
Health status	50.5 (23.9)	49.4 (18.2)	0.86
Type of arthritis *n* (%)^b^			
Inflammatory arthritis	3 (13.0)	5 (15.6)	
Rheumatoid arthritis	12 (52.2)	22 (68.8)	
Psoriatic arthritis	8 (34.8)	4 (12.5)	
Other type of arthritis	0	1 (3.1)	0.22
Medication type *n* (%)^b^			
Not on any DMARDs	6 (26.1)	6 (18.8)	
Mono/combination therapy	11 (47.8)	18 (56.2)	
Biologics (anti-TNF)	6 (26.1)	8 (25)	0.77
Marital status *n* (%)^b^			
Married/living with partner	17 (73.9)	23 (71.9)	
Single	4 (17.4)	5 (15.6)	
Divorced/separated	2 (8.7)	3 (9.4)	
Widow/widower	0	1 (3.1)	0.86
Higher educational attainment^b^			
No	5 (21.7)	4 (12.5)	
Yes	18 (78.3)	28 (87.5)	0.36
Occupational class^b,f^			
Manual	9 (39.1)	10 (31.2)	
Non-manual	14 (60.9)	22 (68.8)	0.54
Type of work^b^			
Full time	12 (52.2)	21 (65.6)	
Part time	11 (47.8)	11 (34.4)	0.32

a Differences between means were tested using two-sample *t*-tests; figures represent mean (SD).

b Differences between groups were analysed using chi-squared tests (*X*^2^). Figures represent *n* (%).

c Physical and mental health component summary were scored using a quality metric scoring algorithm. Scores presented are normalised to the US general population with a mean of 50 and a standard deviation of 10. Scores less than 50 can be interpreted as below the US general population.

d Functional status/psychological scores calculated as the mean response to the 8/4 items in each subscale. For the functional status score this is calculated based on the response to seven completed items for two participants. Functional status/psychological scores range from 0 to 3, higher scores indicating greater difficulty in each domain.

e VAS scores range from 0 to 100, higher scores indicating greater pain/fatigue/ill health.

f Occupational class is classified using the UK Standard Occupational Classification (SOC2010).

SD: standard deviation; HAQ: health assessment questionnaire; DMARD: disease modifying anti-rheumatic drugs; TNF: tumour necrosis factor; VAS: visual analogue scale

Five participants from each group (*n* = 10) took part in the face-to-face interviews at 9 months follow-up to further discuss the initial themes emerging from the data, and the remaining 21 participants were also followed up by telephone for 9 months closing interviews. Those who took part in the face-to-face interviews (*n* = 10) were not followed up by telephone for closing interviews, as they had already provided an extensive account of their views on the work programme at 9 months, and one patient was lost at follow-up.

### Themes emerging from the qualitative analysis

#### Intervention group: Valued the work rehabilitation intervention received and the therapeutic relationship built with the occupational therapist

Intervention group participants valued the practical advice received during the work rehabilitation programme delivered by occupational therapists. The work rehabilitation interventions within this programme included: tips for understanding their body (such as listening to their bodily pain, tiredness); learning to pace their daily activities for energy conservation; how to use aids and adaptations at work to help with joint protection and pain management (such as ergonomic sitting arrangements, use of adapted mouse for hand pain, how to handle heavy loads), as well as self-management of chronic symptoms (such as pain and fatigue) and advice on potential changes to work routines to increase self-efficacy. All of these were considered by participants as having helped them to stay at work. For example, participants said:

Yes, the advice I got was really helpful, because it made you realise what things did help you for the better and, you know, how to look after yourself a bit more. I learned to listen and appreciate what my body is telling me, you know, things like I take regular breaks to reserve my energy now to keep going and have a better sleep hygiene. (4002: Telephone interview at 6 months)

I’d had problems with my thumb and that and I asked her for a splint, a normal splint and she had a look at it and she said, ‘no, you’ve got acute tendonitis and it’s unstable’. So, she organised an injection with my rheumatologist, which I felt a lot better afterwards. She also advised me to buy a rubber grip to fit my pen, to make it easier to write as I found this extremely painful … This really helped me to cope. (1008: Face-to-face interview at 6 months)

Additionally, the intervention group participants had applied the strategies learned from the work rehabilitation programme, and translated these into their daily lives, adapting the way they approached work. This resulted in having better coping strategies for dealing with day-to-day difficulties. For example:

I have been more aware of trying to really take breaks and listen to my body in terms of not try and always do things, and go at a good pace so that I’m not going to irritate my condition in any way. So now I know I can’t go at a speed as I did before and expect to do it all over again the next day. I know I must slow down and listen to my body. This way I get more done, even though it may take me a little longer to do things. (4006: Closing telephone interview at 9 months)

The benefits of the therapeutic relationship developed with occupational therapists, and its positive impact upon their physical and emotional wellbeing, were discussed, with an emphasis on the therapists’ active listening skills. For example:

The therapist actually listened to me, you know, asked me about my experience of having the RA rather than telling me what I should expect! This put me at ease, I found just talking to her about my problems and her acknowledgement of these made me feel better about it. (6003: Telephone interview at 6 months)

It was such a relief to talk about my work problems. Especially, being told that there is help out there for people struggling at work with pain and fatigue daily, like myself. That I can take control of this. (5001: Face-to-face interview at 6 months)

#### Control group: participants reported no benefits in relation to the written work advice received, and lacked future aspirations to stay employed

Participants in the control group described the continuing negative impacts of IA on their work participation and emphasised their debilitating problems. They reported feeling helpless, and the majority were overwhelmed with anxious thoughts. For example:

Well, because I need more joint replacements, and this will be number six and number seven. I just thought, ‘Oh God’, I just feel like I’m facing, like, probably an endless round of surgery in the years to come. So if that became a reality, where I was constantly having to go off – because I mean, a joint replacement is like a 12 week recovery period – if I constantly had to keep going off to get my joints done again, or new ones done, which I need to get done now, I think I would have to say I’m going to have to go back on long-term sick, because realistically, my employer’s not going to be very happy with having me off more than I’m in work. So yeah, I do worry about losing my job at the moment. (6004: Telephone interview at 6 months)

It appeared that participants were unaware of their rights at work, refrained from disclosing their condition to their employers, and expressed concerns about taking frequent sick leave, anticipating this might result in job loss. For example:

I am worried about losing my job if I keep taking time off, so I’ve not been off, no. I don’t really want to get a bad sick record through work. I know it’s an illness you’ve got, you know, but where things are with employment these days, I mean if you’re off on the sick all the time I think is it going to build me up a bad picture. I can’t afford to lose my job in this climate. I don’t want to tell my boss about what I have and worry him about my performance and all. (5003: Face-to-face interview at 6 months)

They frequently expressed feeling isolated due to fatigue following work, which had an impact on their participation in social and leisure activities. For example:

Being in pain day in and out and feeling tired all the while you have to go to work, it’s depressing. By Friday night I am absolutely knackered and I am not interested in going out and seeing friends, not because I don’t want to, I am simply not able to. (6001: Telephone interview at 6 months)

#### Control group: participants didn’t read the work information pack

A majority of people (27/32) reported not having read the work information pack, which was the only advice received by the participants in the control group. For example:

The booklet? Oh I am done with reading booklets about arthritis, so no, I had not read it. (Control group 6011: Telephone interview at 6 months)

I don’t tend to read written information about my condition, I mean I think I know all I need to know about it already. (Control group 4011: Telephone interview at 6 months)

Those few who had read the booklet could not identify specific information that helped. For example:

I think because the information that’s on there is for people who maybe are a lot more disabled with their rheumatoid arthritis than I am … (Treatment group 4008: Face-to-face interview at 6 months).

I know I probably have read it because I do remember the pack, whether I’ve retained that information is another matter really. I think I’m probably one of them people who would have to see somebody face-to-face to retain the information. (Control group 6003: Face-to-face interview at 6 months)

## Discussion

This study provides an important contribution to the field of rheumatology rehabilitation through presenting the views of employed people with IA about the work rehabilitation programme received, and highlighting the factors which may influence job loss or retention in people presenting with work instability. Findings suggest that employed people with IA who received an occupational therapy-led work rehabilitation intervention found that the practical advice and psychological support received helped them to better cope with their difficulties at work, and increased their confidence in self-management of their condition, resulting in improved self-efficacy. Provision of the written advice pack without the individualised tailored approach to patient education was not sufficient to help people with IA who were at work but struggling due to the impact of their condition on their functional status.

As part of this work rehabilitation programme, all participants were provided with a written work advice pack, but this was the only advice received by the participants in the control group. Despite the fact that this is currently the usual practice in the UK for people with arthritis who have work problems, 84% of participants taking part in this study reported that they did not read the written work advice pack received. Those few who reported reading the booklet said that they did not make any changes as a result of reading the work advice pack. Although this behaviour may initially appear inexplicable, literature suggests that information-focused patient education may improve knowledge, but does not necessarily result in a change of behaviour or impact on health status (Hammond, 2008). This perspective was supported by participants’ views in this study, as people from the intervention group were better able to cope with their condition at work by incorporating strategies learned from the one-to-one, tailored work rehabilitation intervention received from the occupational therapist. These strategies were developed following the occupational therapists’ use of WES-RC to identify and prioritise work problems, and a collaborative approach was taken to ensure potential solutions were suitable for the patient’s abilities, environment, and beliefs. As the literature suggests, for the required behaviour change, a tailor-made, individualised, and collaborative practice is essential for the implementation and evaluation of the learned strategies in practice (Hamnes et al., 2011; Kristiansen et al., 2012).

Participants in the control group continued to struggle to cope with the demands of their jobs, and found it difficult to deal with increasing symptoms of IA such as morning stiffness, pain, and fatigue. Indeed, these symptoms are identified as the most common problems affecting the ability to work in people with IA (Katz, 2005; Strand and Khanna, 2010). For instance, in a survey of employed people with RA (*n* = 274), nearly three quarters of participants reported that impaired morning function significantly affected their job, with nearly half reporting having missed time from work in the past six months as a result of this (da Silva et al., 2011). Participants in the intervention group also discussed experiencing difficulties at work in relation to the chronic symptoms of IA; however, they were better equipped to cope with them due to the timely and effective implementation of self-management strategies and the on-going psychological support received from the therapist during their one-to-one work rehabilitation programme, which was tailored specifically to their needs. Previous studies have also identified the need for psychological support in people with IA (Dures et al., 2016; Van der Meer et al., 2011). Dures et al. (2016) conducted a survey of 1210 people with IA in the UK (74% women; mean age 59 years (SD 12.7); patient global 5 (2.3); disease duration < 5 years (41%), 5–10 (20%), >10 (39%)). The results of this study suggested that the demand for psychological help and support is high amongst working people with IA. However, the authors added that fewer than one in four patients with IA were approached about social and emotional issues by a rheumatology professional, despite the fact that 46% would have liked the opportunity to discuss these issues with them (Dures et al., 2016). This clearly identifies the need for health professionals to provide psychological support to working people with IA.

Participants in the control group also mentioned the spill-over effect of difficulties faced at work on participants’ social and leisure activities. Reports of spending the evenings and weekends resting to cope with work, and using annual leave allowance for sick leave to conceal the impact of IA on their work performance from their colleagues and employers, were frequent. This resulted in isolation from family and friends and consequent low mood. Certainly, loss of valued life activities is commonly associated with reduced self-esteem, life satisfaction, perceived health status, and higher levels of depression and pain in RA (Katz, 2005; Katz and Morris, 2007; Katz et al., 2006). High levels of fatigue and low mood experienced without any formal psychological support may explain the lack of motivation to access health and social care by the control group participants. Only one participant in this group reported having read the written advice pack, but was unable to recall whether she found the booklet of help, and admitted that she disposed of it immediately after reading. Schwartz et al. (2014) put forward that symptoms associated with chronic pain, such as fatigue and depression, are characterised by reduced motivation to initiate or complete goal-directed tasks. Thus, the apparent lack of interest in reading written information, or lack of action to change health behaviour upon reading it, may be explained by a person not having the tools to be able to cope with the chronic pain and fatigue they experience.

The participants in the intervention group reported that they were better able to cope with their condition following the intervention, and had a positive outlook about being employed in the future. The authors propose that this is likely to be the result of the self-management education provided by the occupational therapists. Being able to self-manage is an important skill for working people with IA, as they do not often have the time or resources to seek health professionals’ help. Self-management education in IA involves providing strategies to manage pain and fatigue, increase physical activity, pacing, and implementation of good sleep hygiene (Dziedzic and Hammond, 2010). It is acknowledged that self-management education should include cognitive-behavioural approaches for patients to make behavioural changes (Hammond, 2003); therefore, providing a written information booklet to people experiencing work instability is unlikely to be an effective strategy to tackle work disability in people with IA.

Although this study is an important addition to the work rehabilitation literature for people with rheumatic conditions, it is not without limitations. As this qualitative study was nested within a pilot feasibility RCT, recruitment was conducted via a small number of trusts across the North of England, thus, in terms of socio-demographic characteristics, the study population may not be representative of the wider UK population. Although generalisability is not the main purpose of qualitative research, socio-demographic and geographical differences across populations may result in altered health-seeking behaviour (Sheeran and Abraham, 1996). For example, the approach of people from inner city London to the written work information pack may be different from those living in the rural areas of Northern England due to differences in educational attainment.

Data collection in this study was predominantly through telephone interviews. This method of data collection has been subject to criticism in qualitative research due to the potential distractions associated with participants’ own environments (McCoyd and Kerson, 2006), lack of visual cues and non-verbal communication (Garbett and McCormack, 2001), and the interviews being relatively shorter compared to face-to-face interviews (Sweet, 2002). These factors are thought to compromise the quality or the richness of the data obtained (Novick, 2008). In order to reduce the impact of the potential limitations associated with telephone interviews, participants were contacted in advance to arrange a convenient time to conduct the interview, informed that the telephone call will take up to a half an hour, and politely reminded to ensure that their environment should preferably be free of distractions during the interviews. Participants were also given the opportunity to ask any questions they may have and, in advance of the telephone interview, were sent an information sheet to outline the purpose of the interview and how the information provided in these interviews may be used for research purposes. This helped participants to prepare for the interview and provided the chance to develop a rapport between the participant and the researcher in advance of the semi-structured interview. In addition, 10 participants were involved in the face-to-face interviews following the telephone interviews at 6 months to ensure that participants’ views of the work rehabilitation or the work advice received in this study were fully explored.

Furthermore, despite the methodological precision to minimise bias in this study, some degree of bias is nearly always present in research (Panucci and Wilkins, 2010), particularly as qualitative research is criticised for employing subjectivism, which may result in researcher bias and prevent the researcher from considering the participant’s psychological reality. However, the authors support the theory which opposes the post-modernistic view that subjectivity interferes with objective interpretations of the data. Instead, the authors take the view that subjective processes enable a researcher to immerse themselves in the analyses and objectively study complex data to elucidate the truth (Ratner, 2002).

This qualitative study was nested within a pilot feasibility RCT to identify the participants’ views of the work rehabilitation programme or advice received. The findings of this qualitative study will be used to further support the results of the pilot feasibility trial and inform the design of the future definitive RCT to establish the effectiveness of an occupational therapy-led work rehabilitation intervention to help people with IA to stay at work.

## Conclusion

Working people with IA, receiving a work rehabilitation intervention from rheumatology occupational therapists, considered that it helped them to manage their condition at work and understand their employment rights. Compared to those only receiving standard written work advice in the control group, participants in the intervention group were better able to cope with common symptoms of IA such as pain, fatigue, and stiffness, and were more optimistic about staying employed in the future. This study suggests that working people with IA need psychological support from rheumatology professionals to help them cope with the common symptoms of IA and difficulties at work, as well as a tailored work rehabilitation programme which incorporates a cognitive-behavioural approach to self-management education.

## Key findings

- Working people with IA need emotional and psychological support as well as practical self-management education focused on work.
- Written information packs are seldom read by patients with work problems, and do not lead to making changes at work.

## What the study has added

This study is an important addition to both occupational therapy and rheumatology literature, demonstrating that working people with inflammatory arthritis who received an occupational therapy-led job retention work rehabilitation programme specifically tailored for their needs perceived they were able to cope better with their difficulties at work, whilst a written work advice pack was found to be unhelpful.

## Supplementary Material

Supplementary material

Supplementary material

## Supplemental material

The online appendices are available at: http://bjo.sagepub.com/content/by/supplemental-data.

## Note

1 This study was nested within a multi-centre pilot feasibility randomised controlled trial (RCT) (ISRCTN76777720: DOI 10.1186/ISRCTN7677772), 21 September 2012, International Standard Randomised Controlled Trial Number Register (ISRCTNregistry) ‘Work rehabilitation in inflammatory arthritis – Effectiveness and cost effectiveness of an occupational therapy (OT) job retention intervention: A pilot randomised controlled trial’.

## References

[bibr1-0308022616672666] Allaire S Keysor JJ (2009) Development of a structured interview tool to help patients identify and solve rheumatic condition-related work barriers. Arthritis and Rheumatism 61: 988–995. 19565550 10.1002/art.24610

[bibr101-0308022616672666] Allaire SH, Li W and La Valley MP (2003) Reduction of job loss in persons with rheumatic diseases receiving vocational rehabilitation: A randomised controlled trial. *Arthritis and Rheumatism* 48(11): 3212–3218. 10.1002/art.11256 14613285

[bibr2-0308022616672666] Allaire SJ Niu J Zhu Y (2011) Providing effective early intervention vocational rehabilitation at the community level. Rehabilitation Counseling Bulletin 54: 154–163.

[bibr3-0308022616672666] Baldwin D Johnstone B Ge B (2012) Randomized prospective study of a work place ergonomic intervention for individuals with rheumatoid arthritis and osteoarthritis. Arthritis Care & Research 64: 1527–1535. 22511570 10.1002/acr.21699

[bibr4-0308022616672666] Bansback N Zhang W Walsh D (2012) Factors associated with absenteeism, presenteeism and activity impairment in patients in the first years of RA. Rheumatology 51(2): 375–384. 22179728 10.1093/rheumatology/ker385

[bibr5-0308022616672666] Charmaz KC (2006) Constructing Grounded Theory: A Practical Guide through Qualitative Analysis, London: SAGE.

[bibr6-0308022616672666] da Silva JA Phillips S Buttgereit F (2011) Impact of impaired morning function on the lives and well-being of patients with rheumatoid arthritis. Scandinavian Journal of Rheumatology Supplement 125: 6–11. 21529304 10.3109/03009742.2011.566434

[bibr7-0308022616672666] Dewing KA (2015) Management of patients with psoriatic arthritis. Nurse Practioner 40: 40–46. quiz 46–47. 10.1097/01.NPR.0000461950.23292.18 25710245

[bibr8-0308022616672666] Diracoglu D Baskent A Celik A (2008) Long-term effects of kinesthesia/balance and strengthening exercises on patients with knee osteoarthritis: A one-year follow-up study. Journal of Back and Musculoskeletal Rehabilitation 21: 253–262.

[bibr9-0308022616672666] Dures E Almeida C Caesley J (2016) Patient preferences for psychological support in inflammatory arthritis: A multicentre survey. Annals of Rheumatic Diseases 75: 142–147. 10.1136/annrheumdis-2014-205636 25261572

[bibr10-0308022616672666] Dziedzic K and Hammond A (eds) (2010) *Rheumatology: Evidence Based Practice for Physiotherapists & Occupational Therapists.* Edinburgh: Elsevier.

[bibr11-0308022616672666] Garbett R McCormack B (2001) The experience of practice development: An exploratory telephone interview study. Journal of Clinical Nursing 10: 94–102. . 11820243 10.1046/j.1365-2702.2001.00455.x

[bibr12-0308022616672666] Gilworth G Chamberlain MA Harvey A (2003) Development of a work instability scale for rheumatoid arthritis. Arthritis & Rheumatology 49: 349–354. 10.1002/art.11114 12794790

[bibr14-0308022616672666] Gottlieb A Korman NJ Gordon KB (2008) Guidelines of care for the management of psoriasis and psoriatic arthritis: Section 2. Psoriatic arthritis: Overview and guidelines of care for treatment with an emphasis on the biologics. Journal of American Academic Dermatologists 58: 851–864. 10.1016/j.jaad.2008.02.040 18423261

[bibr15-0308022616672666] Hammond A (2003) Patient education in arthritis: Helping people change. Musculoskeletal Care 1: 84–97. 20217669 10.1002/msc.44

[bibr16-0308022616672666] Hammond A (2004) Rehabilitation in rheumatoid arthritis: A critical review. Musculoskeletal Care 2: 135–151. 17041978 10.1002/msc.66

[bibr17-0308022616672666] Hammond A (2008) Rehabilitation in musculoskeletal diseases. Best Practice Research in Clinical Rheumatology 22: 435–449. 10.1016/j.berh.2008.02.003 18519098

[bibr18-0308022616672666] Hamnes B Hauge MI Kjeken I (2011) ‘I have come here to learn how to cope with my illness, not to be cured’: A qualitative study of patient expectations prior to a one-week self-management programme. Musculoskeletal Care 9: 200–210. 21774066 10.1002/msc.212

[bibr19-0308022616672666] Health and Safety Executive (2015) Work-related musculoskeletal disorder (WRMSDs) statistics, Great Britain, 2015. National Statistics.

[bibr20-0308022616672666] Imboden JB Hellman DB Stone JH (2013) Current Diagnosis & Treatment Rheumatology, 3rd ed USA: McGraw Hill Medical.

[bibr21-0308022616672666] Katz PP (2005) Use of self-management behaviors to cope with rheumatoid arthritis stressors. Arthritis & Rheumatology 53: 939–949. 10.1002/art.21580 16342099

[bibr22-0308022616672666] Katz P Morris A (2007) Time use patterns among women with rheumatoid arthritis: Association with functional limitations and psychological status. Rheumatology 46: 490–495. 16936332 10.1093/rheumatology/kel299 PMC2875174

[bibr23-0308022616672666] Katz PP Morris A Yelin EH (2006) Prevalence and predictors of disability in valued life activities among individuals with rheumatoid arthritis. Annals of Rheumatic Diseases 65: 763–769. 10.1136/ard.2005.044677 PMC1798183 16249225

[bibr24-0308022616672666] Kristiansen TM Primdahl J Antoft R (2012) Everyday life with rheumatoid arthritis and implications for patient education and clinical practice: A focus group study. Musculoskeletal Care 10: 29–38. 22213280 10.1002/msc.224

[bibr102-0308022616672666] Macedo A, Oakley SP, Panayi GS, et al. (2009) Functional and work outcomes improve in patients with rheumatoid arthritis who receive targeted, comprehensive occupational therapy. *Arthritis Care and Research* 61(11): 1522–1530. 10.1002/art.24563 19877106

[bibr103-0308022616672666] Mauthner NS and Doucet A (2003) ‘Reflexive accounts and accounts of reflexivity in qualitative data analysis’. *Sociology*, Vol.37, No.3, pp. 413–431.

[bibr25-0308022616672666] Mythen G Walklate S (2006) Beyond the Risk Society: Critical Reflections on Risk and Human Security, Maidenhead: Open University Press.

[bibr26-0308022616672666] NICE (2016) *National service framework: Long term conditions - Publications - GOV.UK*. Available at: https://www.gov.uk/government/publications/quality-standards-for-supporting-people-with-long-term-conditions.

[bibr27-0308022616672666] Novick G (2008) Is there a bias against telephone interviews in qualitative research?. *Research in Nursing & Health* 31(4): 391–398. 10.1002/nur.20259 PMC3238794 18203128

[bibr104-0308022616672666] O'Cathain A, Hoddinott P, Lewin S, et al. (2015) Maximising the impact of qualitative research in feasibility studies for randomised controlled trials: guidance for researchers. *Pilot and Feasibility Studies* 1: 32. 10.1186/s40814-015-0026-y PMC5154038 27965810

[bibr28-0308022616672666] Panucci CJ Wilkins EG (2010) Identifying and avoiding bias in research. Plastic Reconstruction Surgery 126(2): 619–625. 10.1097/PRS.0b013e3181de24bc PMC2917255 20679844

[bibr105-0308022616672666] Prior Y (2013) *An Epidemiological Study Of Self-Care Restriction and Joint Pain in Community-Dwelling Older People*. A Ph.D. Thesis. Arthritis Research UK, Primary Care Centre, Keele University, UK.

[bibr29-0308022616672666] Prior Y Hammond A (2014a) Work rehabilitation for those with rheumatoid arthritis in the UK: A systematic review. Journal of Rheumatology Occupational Therapy 28: 12–16.

[bibr30-0308022616672666] Prior Y Hammond A (2014b) Do occupational therapy services fulfil the work related needs of rheumatology patients in the UK? Annals of the Rheumatic Diseases 73(Suppl 2): 47. .

[bibr31-0308022616672666] Prior Y Amanna A Bodell S (2015) A qualitative evaluation of an occupational therapy-led work rehabilitation for people with inflammatory arthritis: Perspectives of the therapists and their line managers. British Journal of Occupational Therapy 78: 465–466. 26321786 10.1177/0308022615581312 PMC4538318

[bibr32-0308022616672666] Ratner C (2002) Subjectivity and objectivity in qualitative methodology. Forum: Qualitative Social Research 3(3): 16.

[bibr33-0308022616672666] Schwartz N Temkin P Jurado S (2014) Chronic pain. Decreased motivation during chronic pain requires long-term depression in the nucleus accumbens. Science 345(6196): 535–542. 25082697 10.1126/science.1253994 PMC4219555

[bibr34-0308022616672666] Sheeran P Abraham C (1996) The health belief model. In: Conner M Norman P (eds) Predicting Health Behaviours: Research and Practice with Social Cognition Models, Buckingham: Open University Press, pp. 23–61.

[bibr109-0308022616672666] Sofaer S (1999) Qualitative methods: What are they and why use them? *Health Services Research* 34(5 Pt 2): 1101. PMC1089055 10591275

[bibr35-0308022616672666] Strand V Khanna D (2010) The impact of rheumatoid arthritis and treatment on patients’ lives. Clinical and Experimental Rheumatology 28: S32–S40. 20576223

[bibr36-0308022616672666] Sweet L (2002) Telephone interviewing: Is it compatible with interpretive phenomenological research? Contemporary Nurse 12: 58–63. . 12013519 10.5172/conu.12.1.58

[bibr37-0308022616672666] Uhlig T (2010) Which patients with rheumatoid arthritis are still working? Arthritis Research Therapy 12: 114. 20441606 10.1186/ar2979 PMC2888217

[bibr38-0308022616672666] Van der Meer M Hoving JL Vermeulen MI (2011) Experiences and needs for work participation in employees with rheumatoid arthritis treated with anti-tumour necrosis factor therapy. Disability Rehabilitation 33: 2587–2595. 21671833 10.3109/09638288.2011.582923

[bibr39-0308022616672666] Williams SJ (2014) Is anybody there? Critical realism, chronic illness and the disability debate. Sociology of Health & Illness 21(6): 797–819.

