# Supplementary material for: A qualitative evaluation of occupational therapy-led work rehabilitation for people with inflammatory arthritis: Patients’ views
Source: Br J Occup Ther. 2016 Nov 21;80(1):39–48. doi: 10.1177/0308022616672666 (PMC6097118; doi:10.1177/0308022616672666)
Supplement: Supplementary material [file BJO672666_supp_data_1-3.docx]

**Appendix 1: Interview Schedule for 6 Months Telephone Follow-up**

Participants were asked:

1. Are you still in paid work?

**If yes:**

- 1. In your view, what factors have enabled you to stay in work?

**Prompts** (Only used to elicit participants views further, if needed):

1. Have you made any practical changes?
2. Have you had any discussions with your employer about your condition?
3. What support have you received from your employer to help you stay in work?
4. Do you have any concerns about continuing to work in future?

**If no:**

1.2 In your view, what were the reasons for you stopping work?

**Prompts:**

1. Were you able to discuss your condition with your employer? Were you able to discuss any possible work advice/ changes with them?
2. Are you aware if your company has a health attendance policy? Was it helpful for you?
3. Are there any other factors, which contributed?

1.3 Do you plan to return to employment in future? (In order to identify if this was a redundancy, taking early retirement and if likely to be a temporary or permanent job loss)

**Appendix 2: Interview Schedule for 6 Months Face-to-Face Follow-up Interviews**

Participants were asked:

1. What are your views about the work assessment and support provided by the Occupational Therapist [for those in the intervention group] and/ or the written work advice mailed to you [both for those in the intervention group and the control group]?
2. What types of assistance do you recall receiving from the therapist [or do you recall seeking] as a result of the intervention/ or what kind of help the written work advice booklet has provided?
3. What are your thoughts on continuing to work in the future [for both groups]?

**Prompts:**

1. How did you find these different elements?
2. Was there any advice/ assistance that you consider were unnecessary? If yes, why?
3. Was there any advice/ assistance you would have liked to receive but did not? If yes, why?
4. Was the final outcome of the work programme [either the intervention received from the OT or the written work advice] right for you?
5. Are there any other comments you would like to make about the work support provided?

**Appendix 3: Interview Schedule for 9 Months Telephone Follow-up**

Participants were asked:

1. Do you think the advice/ intervention you have received in this study made any difference to your life?
2. Thinking back to the intervention (For the intervention group);
   1. Was the length of time between the first contact and getting the intervention was appropriate?

Prompts:

- - 1. How did you go about to arrange the time and place to meet with the therapist?
    2. Where did you meet with the OT/ Were you offered a choice in most appropriate time and place to meet?
    3. What would have been the best time and place for you?

1. What did you think about the information pack you received (Booklet only group)?

Prompts:

- 1. How helpful was the booklet (i.e. written work advice)?
  2. Did you take any action as a result of anything you read in the booklet to help you at work?

1. Have you used annual leave for sickness absence in the past 3 months?

Prompts:

- 1. If yes, why? – can you remember how many days?
  2. If no, have you ever?

1. How would you describe your work colleagues’ attitude to your arthritis?
2. Have receiving work advice helped any other areas outside of your work life?
