# Supplementary material for: A qualitative evaluation of occupational therapy-led work rehabilitation for people with inflammatory arthritis: Patients’ views
Source: Br J Occup Ther. 2016 Nov 21;80(1):39–48. doi: 10.1177/0308022616672666 (PMC6097118; doi:10.1177/0308022616672666)
Supplement: Supplementary material [file BJO672666_supp_data_4.docx]

Patients in the intervention group have not read the booklet but were better informed of their rights at work and strategies to help

Someone to really listen

Emphasise on the +ve rather than the -ve

Practical strategies

Job accommodations

Their rights at work

Refrained from disclosure

Limited coping skills

Lack of recognition & psychological support

Helplessness in managing their condition

Lack of pain, and fatigue management strategies

Worried about taking sick leave

Preconception about employer’s views

Unaware of their rights at work

Not being able to meet the demands of their job

Not read the work advice pack

Don’t remember reading the work advice pack

Too many written information is available and no longer reads them (i.e. fed up with them)

Read the work advice pack but did not take any action
